# A systematic review of infectious illness Presenteeism: prevalence, reasons and risk factors

**DOI:** 10.1186/s12889-019-7138-x

**Published:** 2019-06-21

**Authors:** R. K. Webster, R. Liu, K. Karimullina, I. Hall, R. Amlôt, G. J. Rubin

**Affiliations:** 1 Department of Psychological Medicine, Institute of Psychiatry, Psychology and Neuroscience, King’s College London, London, England; 2 National Institute for Health Research Health Protection Research Unit (NIHR HPRU) in Emergency Preparedness and Response, King’s College London, London, England; 3 School of Mathematics, The University of Manchester, Manchester, England; 4 Behavioural Science Emergency Response Department Science and Technology, Public Health England, Porton Down, England; 5 Faculty of Philosophy, University of Oxford, Oxford, England

**Keywords:** Presenteeism, Working while ill, Infectious illness, Flu, Influenza-like-illness, Prevalence, Risk factors

## Abstract

**Background:**

Workplace presenteeism is common and leads to the spread of infectious diseases. Previous reviews have focused on presenteeism in relation to general physical or mental ill health. In this systematic review we identified the prevalence of, and reasons and risk factors for, presenteeism in relation to an infectious illness.

**Method:**

We searched Medline, Scopus, Web of Science, PsycINFO and PsycARTICLES with terms relating to infectious illnesses and presenteeism at the work place or school; reference lists of relevant articles were also hand-searched.

**Result:**

Our search yielded 3580 papers after deduplication. After title, abstract and full text screening, 23 papers reporting on 24 studies were included. Twenty-three studies were cross-sectional studies and one was prospective. The quality of included studies was relatively poor due to problems such as sampling and non-response bias. Presenteeism prevalence ranged from 35 to 97%. Self-reported reasons for presenteeism fell into three main themes: 1. Organisational factors (organisational policy, presenteeism culture, disciplinary action), 2. Job characteristics (lack of cover, professionalism, job demand), and 3. Personal reasons (burden on colleagues, colleague perceptions, threshold of sickness absence and financial concerns). Statistical risk factors fell into four themes: 1. Sociodemographic, 2. Health, 3. Influenza-related behaviour, and 4. Employment characteristics. Most of the risk factors had insufficient evidence to allow us to draw any firm conclusions, and evidence regarding gender and age was inconsistent. The risk factor with the most consistent findings concerned occupation type, suggesting that those who worked in the healthcare sector, and specifically physicians, were at a higher risk of infectious illness presenteeism.

**Conclusion:**

Infectious illness presenteeism is common. To address the public health consequences, organisations should focus on promoting a positive working culture and developing sickness absence policies that reduce presenteeism. Further research is needed in non-health sector organisations and schools to identify risk factors related to different organisations, which can then be used to tailor interventions at the organisational and individual level.

**Electronic supplementary material:**

The online version of this article (10.1186/s12889-019-7138-x) contains supplementary material, which is available to authorized users.

## Background

Presenteeism is most commonly defined as people who attend work in spite of their illnesses [1]. Compared to absenteeism, the concept of presenteeism is relatively under-studied. However, presenteeism is a global phenomenon that is common among employees of all levels and has been suggested to cause a greater loss to an organisation than the costs attributed to absenteeism [2] through productivity loss [3], and future poor health and sick leave [4]. Although presenteeism has most often been studied in employees, it can also be seen in non-workplace environments, for instance universities, schools and nurseries [5].

Studies have shown that over 60% of employees have attended work while sick, rising to 90% in some studies of occupations such as physicians [4]. Antecedents of presenteeism may include feelings of being irreplaceable, a high workload, not being able to afford to take time off, perceiving presenteeism as an organisational norm, or perceiving that you are not sick enough to justify time off work [1, 6]. However, most research has focused on presenteeism due to chronic conditions [3]. Presenteeism with acute infectious illnesses such as influenza can arguably present more problems to organisations due to the possibility of workplace epidemics.

Employees who continue to work despite having symptoms of an infectious illness pose a risk to others, especially to people who are vulnerable to diseases, such as patients, the elderly, and children [7–9] . This may be particularly true for health or social care professionals working with vulnerable populations [10, 11]. In the worst case scenario, presenteeism can even contribute to pandemics, as illnesses circulate within workplaces and education settings [12]. The problems associated with presenteeism are certainly recognised within the general public: a representative survey by Canada Life Group [13] reported that 82% of UK workers say they have become ill as a result of a colleague coming into work when they are unwell.

Given its public health importance, in this review we sought to summarise the prevalence of, self-reported reasons and statistical risk factors for presenteeism associated with an infectious illness in workplaces or educational / childcare settings. Our intention was to assist in highlighting possible public health approaches to presenteeism, avenues for future research, and to identify areas where there is potential to develop interventions to reduce presenteeism.

## Methods

The reporting of this review adheres to the standards for the Preferred Reporting Items for Systematic reviews and Meta-Analyses [14].

### Search strategy

KK and RL carried out preliminary work testing a variety of different search strategies, to balance both specificity and sensitivity. These were finalised in discussions with GJR and IH. Our final search strategy used terms and associated words for ‘acute infectious illness’ and ‘presenteeism’, joined by the AND function. A copy of our search strategy in MEDLINE is included as Additional file 1. The search strategy was modified for each specific database due to differences in MeSH terms, boolean operators and wildcards. Where possible, searches were limited to articles published in the English language and excluded review articles.

### Searches

The following electronic databases were searched with the predefined search strategy: Web of Science, Scopus, and OvidSp (Medline, PsycINFO, and PsycARTICLES). Web of Science and Scopus were included for their cover of the sciences and social sciences, and for the fact that the two resources together complement each other, as neither of them are all inclusive. OvidSp was chosen for its cover of health science journals, and also for its inclusion of the database PsycINFO, and PsycARTICLES.

### Review process

KK and RL tested the screening process for one database prior to the full database search. This was to ensure consistency in the screening process and clarify any uncertainties about whether studies met the inclusion criteria or not. RW carried out the full search on 12th October 2018 and initial electronic searches from the different databases were combined using EndNote with duplicates identified and deleted. First, the titles and abstracts were screened for mentions of an empirical study examining presenteeism relating to an infectious illness. If it was not clear from the abstract, the study was taken to full text review. All full text versions of papers that remained potentially relevant were screened in relation to the exclusion/inclusion criteria. Those papers that met the inclusion criteria also had their reference section manually searched for any other potential studies that could be included.

### Selection criteria

Studies were eligible for inclusion in this review if they meet the criteria as outlined below:

- ***Population.*** Human population, any age.
- ***Exposure.*** Presenteeism relating to an infectious illness. Presenteeism must be defined as going to work or school while ill.
- ***Outcome.*** The study reported data on prevalence of people attending work or university / school / childcare with an infectious illness, OR the study reported data on risk factors associated with attending work, or university / school / childcare with an infectious illness OR the study reported data on self-reported reasons given for attending work, or university / school / childcare with an infectious illness.
- ***Study design.*** Both qualitative and quantitative studies were eligible. Quantitative studies could be of any design. Articles that did not report on original data, e.g. review articles were excluded.
- ***Other limiters:*** Published in the English language.

### Data extraction

Data from the final set of studies were extracted by RW using a data extraction table which was developed for this systematic review. Data extracted included citation, country of study, study design, main characteristics of participants (sample size, mean age, % male), occupation or industry, illness, and results regarding prevalence of presenteeism of those with infectious or suspected infectious illness, and/or risk factors associated with presenteeism and/or reported reasons for presenteeism.

### Quality assessment

The quality of all eligible studies was assessed using appropriate quality assessment tools for the relevant study designs. These included the CASP critical appraisal tool [15] for qualitative and cohort studies, while the Mixed Method Appraisal Tool (MMAT) [16] was used to assess the quality of other quantitative studies. Instead of using the original yes/ can’t tell/ no answers in checklists, slight adaptation with a final category of “low”, “unclear” or “high” risk was used for each question. Answers “yes”, “can’t tell” and “no” correspond to “low risk”, “unclear risk” and “high risk”, respectively.

### Data synthesis and analysis

Because of the expected heterogeneity in study designs and outcomes, we did not plan for any meta-analyses and instead used a narrative synthesis. There is no general consensus on the best way to carry out a narrative synthesis for systematic reviews [17]. As such we decided to use a weight of evidence approach in order to consider the quality of the studies alongside the results in order to assess the strength of evidence of their findings.

## Results

### Search results

**Fig. 1:**
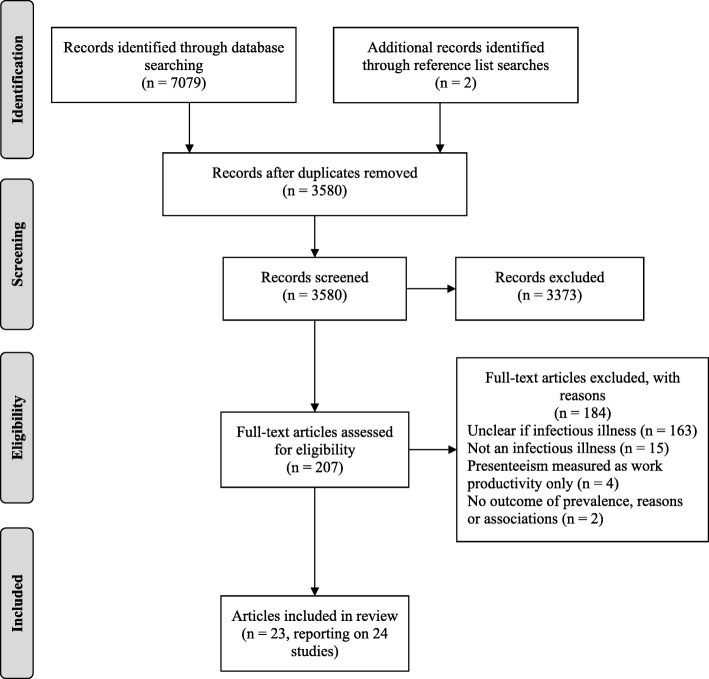
Flow diagram of the screening process and reasons for exclusion

The search yielded a total of 7079 papers, with an additional two papers identified through reference list searches. After removing duplicates, 3580 papers remained. Screening titles and abstracts resulted in 207 papers being taken forward to full text review. Of these,163 papers were excluded for not making it clear if they were measuring presenteeism with regards to infectious illnesses or not, 15 papers were excluded for not measuring presenteeism as a result of an infectious illness, 4 were excluded for measuring presenteeism as changes in work productivity/performance only, and 2 were excluded for not including an outcome of presenteeism prevalence or reasons for, or associations with, presenteeism. As a result, 23 papers were included in the systematic review, one of which reported on two studies [18] and is referred to as study 1 or 2 where necessary. See Fig. 1 for a flow diagram of the screening process and reasons for exclusion.

### Study characteristics

Out of the 24 studies, 23 were cross-sectional, of which 20 were survey based, two were medical record reviews over a certain period and one was qualitative involving individual interviews. The remaining study was prospective involving monthly surveys completed by participants. While most of the studies were conducted in America (*n* = 11), seven were conducted in Europe (United Kingdom, Norway, Portugal, Poland), and three were conducted in Canada and New Zealand. The sample size of included studies ranged from 31 to 550,360. Participant ages ranging from 18 to 97 years old, and the percentage of male participants ranging from 0 to 64.4%. While 16 studies focused on employees from the health care sector, five focused on employees from different organisations, one solely focused on medical students, one sampled participants from the general population of America, and one focused on parents of pre-school children. Ten studies measured presenteeism with regards to influenza-like-illness, seven to respiratory tract infections, five to infectious illnesses in general, and two to symptoms of infectious illnesses. All studies apart from one included an outcome of infectious illness presenteeism prevalence, 12 assessed associations between baseline variables and infectious illness presenteeism, and 10 assessed participants’ reasons for infectious illness presenteeism. See Table 1 for full study characteristics.

**Table 1:** Summary of study characteristics

Reference	Country	Study design	Sample (N, age, % male)	Illness	Outcome(s)
Ablah 2008 [19]	America	Cross-sectional survey	Employees from organisations represented at a Pandemic Influenza Workgroup (1485, < 30- > 60, 28)	ILI	Prevalence, associations
Bhadelia 2013 [20]	America	Cross-sectional records review	HCWs at a tertiary care centre with ILI and tested for influenza (352, 21–68, 25)	ILI	Prevalence, associations
Bracewell 2010 [21]	New Zealand	Cross-sectional survey	Hospital clinical staff (224, < 25- > 55, 19)	Infectious illnesses	Prevalence, reasons, associations
Carroll 2016 [22]	United Kingdom	Cross-sectional interview	Parents of pre-school children (3, 26–47)	RTI	Reasons
CDC 2004 [23]	America	Cross-sectional survey	Noninstitutionalized U.S. civilian adults (2231, 18–97, 48.7)	ILI	Prevalence
Chambers 2017 [24]	New Zealand	Cross-sectional survey	Senior physicians and dentists (1806, 20- > 60, 59)	Infectious illnesses	Prevalence, associations
Chiu 2017 [25]	America	Cross-sectional survey	HCPs during 2014–15 influenza season (1914, 18- > 50, nr)	ILI	Prevalence, reasons, associations
de Perio 2014 [26]	America	Cross-sectional survey	School employees (412, 22–71, 18)	ILI	Prevalence, reasons, associations
Gudgeon 2009 [27]	Canada	Cross-sectional survey	Medical students, surgical residents and staff physicians (668, nr, nr)	RTI	Prevalence, reasons, associations
Jena 2012 [28]	America	Cross-sectional survey	Resident physicians (150, nr, nr)	ILI	Prevalence, reasons, associations
Juszczyk 2018 [29]	Poland	Cross-sectional records review	Patients who were professionally active, employed, or running their own business (550,360, 19–64, 38.1)	RTI	Prevalence
Kobayashi 2016 [30]	America	Cross-sectional survey	Staff members at a skilled nursing facility (162, nr, nr)	RTI	Prevalence
LaVela 2007 [31]	America	Cross-sectional survey	HCWs caring for persons with spinal cord injuries (820, < 25- > 65, 26.71)	RTI	Prevalence, associations
Martinez 2012 [32]	Portugal	Cross-sectional survey	Nurses from a major public hospital (296, M = 35.7, 27.7)	RTI	Prevalence
Mitchell 2017 [33]	Canada	Cross-sectional survey	Resident physicians (323, nr, 20.1)	Symptoms of infectious illness	Prevalence, associations
Mossad 2017 [34]	America	Cross-sectional survey	HCPs caring for transplant and internal medicine patients (286, Me = 35, 28)	ILI	Prevalence, associations
Perkin 2003 study 1 [18]	United Kingdom	Cross-sectional survey	Junior doctors (81, nr, 56.8)	Infectious illnesses	Prevalence, reasons
Perkin 2003 study 2 [18]	United Kingdom	Cross-sectional survey	Junior doctors (110, nr, 60.0)	Infectious illnesses	Prevalence, reasons
Rebmann 2016 [35]	America	Cross-sectional survey	School nurses (133, < 40- > 61, 0.8)	ILI	Prevalence, reasons
Rosvold 2001 [36]	Norway	Cross-sectional survey	Physicians (1015, M = 42.3, 57)	Infectious illnesses	Prevalence
Rousculp 2010 [37]	America	Prospective monthly survey	Employees from 3 large US employers (793, M = 40.7, 64.4)	ILI	Prevalence, associations
Tan 2014 [38]	New Zealand	Cross-sectional survey	Tertiary care hospital physicians (328, nr, 55)	ILI	Prevalence
Veale 2016 [39]	Canada	Cross-sectional survey	Medical students (549, nr, nr)	Symptoms of infectious illness	Prevalence, reasons
Whysall 2018 [40]	United Kingdom	Cross-sectional survey	Employees of a large UK Utilities organisation (316, nr, nr)	RTI	Prevalence

Note: *ILI* influenza-like-illness, *RTI* respiratory tract infection, *HCPs* health care professionals, *HCWs* health care workers, *nr* not reported, *M* mean, *Me* median

### Quality assessment

**Fig. 2:**
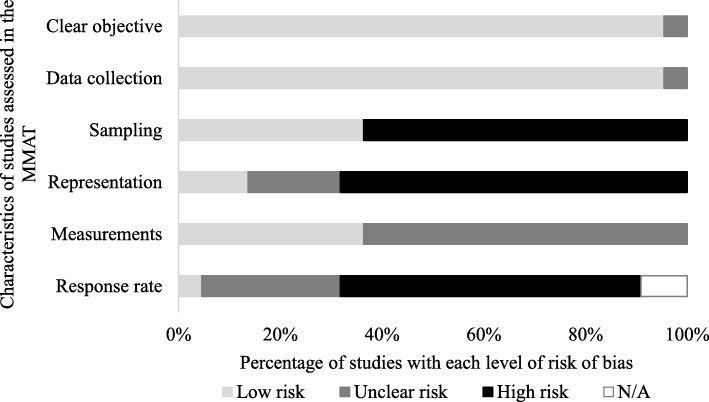
Quality of cross-sectional quantitative studies. *MMAT = Mixed methods appraisal tool

The overall quality of the 22 cross-sectional quantitative studies was poor (see Fig. 2). The majority of studies provided clear objectives for which the data collected was appropriate in addressing those objectives. However, sampling methods were poor and prone to selection bias as the participants were often picked from particular segments within the targeted population, or the authors failed to justify the criteria used for selection. A large proportion of the studies were not representative due to poor sampling strategies and/or small sample size. Only some studies used standardised measurements, the rest did not elaborate on measurement items, making it unclear as to their suitability. The majority of studies had a low (below 60%) response rate and as such were at heightened risk of nonresponse bias. Two studies were medical record reviews therefore response rate was not applicable.

Quality was mixed for the two remaining studies that were prospective or qualitative in design. Carroll, Rooshenas [22] had clear aims and appropriate methodology and design, however there was a high risk of bias for recruitment as the sample was a select group of parents and not representative of the target population. In addition, more detail was needed on the data analysis. Similarly, Rousculp, Johnston [37] had a high risk of bias for recruitment as the sample was not selected to be representative of the target population, and there was a lack of description of both the measures used and drop-outs.

### Prevalence of presenteeism

Twenty-three studies reported prevalence of presenteeism of those with infectious illness, all measuring work place presenteeism, apart from one study which included work place and school presenteeism in the prevalence score [23], and another which measured placement presenteeism in medical students [39]. Although all measures on presenteeism were conceptualized as going to work or school while ill, there were some discrepancies between measurements. For instance, while seven of the studies used the past 12 months as a cut-off point in the survey question, other studies used different time lines varying from 2 to 6 months, years, the length of an influenza season, or without any cut-off point. Overall presenteeism prevalence ranged from 35 to 97%, and for studies of participants who worked in the healthcare sector this was 37 to 97%. Studies of other occupational settings reported 35 to 88.6% presenteeism, and the representative survey of US adults which reported both work place and school presenteeism reported a prevalence of 82.7% in the past five months [23]. Carroll, Rooshenas [22] did not provide an outcome of presenteeism prevalence for pre-school children. See Table 2 for the results of individual studies.

**Table 2:** Prevalence of, reasons for and associations with infectious illness presenteeism

Reference^quality^	Prevalence of presenteeism of those with infectious illness	Reasons for presenteeism	Factors tested for associations with presenteeism (significant associations in bold*)
Ablah 2008^a,b^ [19]	61% (to date)		**Gender (male), age (younger), ILI presenteeism intention (yes), occupation (HCWs and health educators)**
Bhadelia 2013^a,b^[20]	65% (past 12 months)		Occupation
Bracewell 2010^b,c^ [21]	48.7% (past 12 months)	1. Did not want to increase workload of others; 2. No replacement available; 3. Increased burden of work once returned; 4. Not sick enough; 5. Pressure from work; 6. Did not want to cancel clinics; 7. Unwell during days off; 8. Could not cancel clinics; 9. Financial stressors; 10. No more sick leave/ sick days; 11. Concerns about job security	Gender, age, health, dependents, amount of work left undone if absent, hours worked, job satisfaction, **occupation (physician)**
Carroll 2016^a^[22]		1. Nursery fees paid in advance; 2. Alternative child care is an extra cost; 3. Colleagues perceptions if absent from work; 4. Family/ friends are often working; 5. Nursery payment reliant on work income); 6. No guidance to say child cannot be sent into nursery with RTI; 7. No alternative care options	
CDC 2004^b,c^[23]	82.7% (past 5 months)		
Chambers 2017^c^[24]	75% (past 12 months)		**Gender (female), age (younger), number of presenteeism days**, length of time in profession, host of district health board
Chiu 2017^a^[25]	41.4% (during influenza season)	1. Could still perform job duties; 2. Not feeling bad enough to miss work; 3. Did not think it was contagious; 4. Professional obligation to co-workers; 5. Difficult to find cover; 6. Not afford to lose pay; Employer expects staff to work while ill; 7. Risk of being penalised by employer; 8. Professional obligation to patients; 9. Did not have paid sick leave; 10. No one in workplace said to stay home; 11. Missed too much work already this year	Age, patient type, professional/clinical status, length of time in job, **occupation (physician, pharmacist), work setting (hospital), vaccinated during influenza season (yes)**
de Perio 2014^c^[26]	77% (since start of school year)	1. Professional obligation to students; 2. Did not think it was contagious; Difficult to get or prepare for a substitute; 3. Might be penalized by employer; 4. Professional obligation to co-workers	Gender, age, household with children, occupation, workplace, employment status,asthma, diabetes, **healthy immune system**
Gudgeon 2009^a,b,c^[27]	48–60% depending on occupation (nr)	Students: 1. Cared about opinions and impressions of others; 2. Doctors note is required but is often difficult to obtain. Physicians/Residents: 1. Concern over delivery of patient care; 2. Patient impact of rescheduling procedures	**Occupation (physician)**
Jena 2012^a,b^[28]	51% (past 12 months)	1. Did not want to force colleagues to cover; 2. Responsible for patients’ care; 3. Colleagues would think they were “weak”; 4. Pressured to repay colleagues for coverage	Gender, training year
Juszczyk 2018 [29]	35% (average for all RTI infections, in a period of 14 months)		
Kobayashi 2016[30]	53.7% (during 3-month period)		
LaVela 2007^a,b,c^[31]	86% (during influenza season)		**Perceptions of institutional control measures (no droplet precautions, no restriction of staff movement, contact between ill patients and other patients not restricted, no infection control measures)**, influenza and vaccine related behaviour
Martinez 2012^a,b,c^[32]	8.1 days a year attended work with infection (nr)		
Mitchell 2017^a,b,c^[33]	59.1% (past 2 months) 97% (during study period)		Training year
Mossad 2017^a,b,c^[34]	92% (during influenza season)		**Gender (female), age (younger),** patient type
Perkin 2003^a,b^ study 1 [18]	84.9% (1993, past 6 months)	1. Consultant pressure; 2. Colleagues must do extra work; 3. Did not influence capacity to work; 4. No risk of transmission	
Perkin 2003^a,b^ study 2 [18]	63.2% (2001, past 6 months)	1. Consultant pressure; 2. Colleagues must do extra work; 3. Did not influence capacity to work; 4. No risk of transmission	
Rebmann 2016^a,b,c^ [35]	42.1% (past 3 years)	1. Care provider cleared them for work; 2. Illness not severe; 3. No one to cover the work; 4. Risk falling behind; 5. Feel pressured by colleagues or supervisors; 6. No paid sick leave; 7. Worried about getting fired	
Rosvold 2001 [36]	52.8% (past 12 months)		
Rousculp 2010^a^[37]	88.6% (past 6 months)		**Sick leave policy (can’t work from home)**
Tan 2014^c^[38]	49% (past 12 months)		
Veale 2016^a,b,c^[39]	37% (during a clerkship rotation ~ 6 weeks)	1. Must make up the time; 2. Fear of poor evaluation; 3. Sickness not severe; 4. Need the experience; 5. No one to cover; 6. Observed others working while ill; 7. Did not think it was infectious; 8. Did not want to get a doctors’ note; 9. Not sure if should stay home/right thing to do; 10. Pressure from physicians	
Whysall 2018^a,b,c^ [40]	59% (past 12 months)		

Note: *HCWs* health care workers, *ILI* influenza-like-illness, *RTI* respiratory tract infection

^a^ = high risk of sampling bias, ^b^ = high risk of non-representation, ^c^ = high risk of non-response bias

* *p* < .05

### Reported reasons for presenteeism

Ten studies reported the reasons participants gave for infectious illness presenteeism. Reported reasons were similar in nature and could be grouped into different over-arching themes, namely, organisational factors, job characteristics, and personal reasons (Table 2 for individual results).

#### Organisational factors

##### Organisational policy

Five studies reported factors relating to organisational policy as reasons for presenteeism. Three studies reported that working while ill was due to participants not having paid sick leave or no more available sick leave [21, 25, 35]. Veale, Vayalumkal [39] reported that participants did not have clear guidelines from their organisation and so were unsure of the right thing to do regarding staying at home or going into work. In the final study parents reported that their nurseries did not have any guidance on whether children can or cannot attend nursery with an RTI [22].

##### Presenteeism culture

Five studies shed light on the fact that presenteeism in organisations can be a social norm, embedded within the organisational culture. For example, Perkin, Higton [18], Bracewell, Campbell [21], Rebmann, Turner [35], Veale, Vayalumkal [39] reported participants feeling pressure from colleagues to turn up to work when ill, especially when they observed other colleagues doing so. Some participants explicitly stated that their employer expected them to work when ill, and that none of their colleagues suggested they should go home when they turn up to work ill [25].

##### Disciplinary action

Respondents from five studies were concerned that taking sick leave might lead to disciplinary action. Worries of losing their job was the major concern, while fear of getting into trouble, receiving a poor evaluation or being penalized, and being anxious about job security were also reported [21, 25, 26, 35, 39].

#### Job characteristics

##### Lack of cover

Respondents in six studies reported attending work while ill due to the lack of cover available, as temporary workers were usually unavailable or difficult to find [21, 25, 26, 35, 39]. Similarly, for parents’ deciding whether to take their child to nursery when ill, reasons often reported included not being able to find alternative care options to look after their child [22].

##### Professionalism

Five studies reported that having a strong work ethic was crucial for participant professions as they had a duty to their patients and colleagues and taking sick leave might jeopardise their reputations. For example, Chiu, Black [25], de Perio, Wiegand [26], Gudgeon, Wells [27], and Jena, Meltzer [28] all reported that participants felt they had a professional obligation to their colleagues, patients and students to turn up to work. As such, respondents who worked in the health care industry were often worried that substitutes were not qualified to perform certain tasks and therefore reluctant to let others handle their clients and felt they could not cancel their clinics due to the potential impact of substitutes or rescheduling on patient care and well-being [21, 27].

##### Job demand

Three studies reported job related demand as a cause for presenteeism. Employees were concerned with the extra workload they might have when returning to work because tasks would be left undone during their absence [21], as such there were fears of falling behind with their work [35] and having to make up the time when they got back to work [39].

#### Personal reasons

##### Burden to colleagues

Participants in three studies revealed that they did not want to burden colleagues with extra workload resulting from their absence and often felt guilty of asking colleagues to cover duties [18, 21, 28]. In addition, one study noted that participants felt they would then have to repay colleagues for covering for them [28].

##### Perceptions from colleagues

Participants in three studies reported concerns about how colleagues might perceive them in the case of sickness absence. They were afraid to be seen as weak [28] and cared about the opinions and impressions of colleagues if they were absent from work [22, 27].

##### Threshold for sick leave

A common theme running through the reasons given for presenteeism was that participants often thought they did not meet the threshold for which they should take sick leave. Respondents in four studies felt that their illnesses were not severe, and they were well enough to work [21, 25, 35, 39]. Similar reasons included that their illness did not influence their capacity to carry out their work duties [18, 25]. Many respondents also believed that their illnesses were not infectious, so they were not a risk to colleagues, patients or students, and therefore chose to attend work [18, 25, 26, 39].

##### Financial concerns

The last theme which fell into personal reasons for infectious illness presenteeism concerned financial worries. Three studies showed that financial stress was a cause for presenteeism. Participants reported that they could not afford the loss of salary due to sick leave as they needed to support the family [21, 25]. Similarly, parental reasons for taking their child to nursery when ill include the fact that nursery fees were paid in advance, these fees were reliant on the income from their work, and that finding alternative child care comes at an extra cost [22].

#### Other reasons

Some motives for presenteeism did not fit comfortably in the above themes. These included medical students finding it hard or too much of an effort to get a doctor’s note in order to be allowed to take sick leave [27, 39], or feeling that they would be missing out on vital experience if they took sickness leave [39]. Another reason given by HCPs concerned the fact that they had already missed too much work in the year already [25].

### Statistical risk factors for presenteeism

Twelve studies tested the association between different variables and infectious illness presenteeism. These were grouped into four main categories: sociodemographic factors, health, influenza-related behaviour and employment characteristics.

#### Sociodemographic factors

##### Gender

Six studies evaluated the association between gender and presenteeism, with inconsistent results. Three studies did not find any significant associations [21, 26, 28]. Two studies found females reported significantly more presenteeism [24, 34], and one study showed a significant association between being male and presenteeism [19]. Given the varying quality of these studies and the inconsistent findings, we considered the evidence for the association between gender and infectious illness presenteeism to be inconclusive.

##### Age

Six studies investigated the relationship between age and presenteeism, of which three of the higher quality studies found no significant associations [21, 25, 26]. The remaining three all found that being younger was associated with higher levels of presenteeism [19, 24, 34]. The age ranges of the sample were slightly smaller in the studies that found no significant effect, so this could have contributed to these findings. However, given the contrasting findings at this point the evidence for the association between age and infectious illness presenteeism is inconclusive.

##### Dependents

Two studies of higher quality found no associations between whether participants had any dependents [21], or a household with children [26] with infectious illness presenteeism.

#### Health

Three studies looked at whether participant health status is associated with infectious illness presenteeism, showing inconsistent effects. Bracewell, Campbell [21] found that self-reported health was not significantly associated with presenteeism. Similarly, in a higher quality study de Perio, Wiegand [26] found that having a chronic condition such as asthma or diabetes was not related to presenteeism. However, participants who had a healthy immune system that was not weakened by illnesses such as cancer or immunosuppressant medication were more likely to report presenteeism [26].

#### Influenza-related behaviour

Influenza-related behaviour was another factor which was included in studies. Ablah, Konda [19] found that intention to go work with an ILI was associated with actual presenteeism. Similarly, Chambers, Frampton [24] found that those who had more presenteeism days in the past, had higher levels of current presenteeism. Chiu, Black [25] found that those who had received their influenza vaccine for that season had higher levels of presenteeism. However, a lower quality study found no significant associations between various influenza related behaviour variables and presenteeism such as receiving the vaccine that season, how often they receive the influenza vaccine, if they recommend it to patients and if they have taken antiviral medications [31]. Therefore, there is some indication of the role that past influenza-related behaviour and future intentions are associated with current presenteeism, but more robust research is needed.

#### Employment characteristics

##### Occupation

Six studies measured the association between occupation and infectious illness presenteeism. Ablah, Konda [19] found that those who worked in the health care sector as opposed to non-health occupations were significantly more likely to engage in presenteeism. Within non-health settings, de Perio, Wiegand [26] found no effect of occupation type in a school setting, or whether their job took place within the school or not. The remaining four studies looked at occupation type within the health care sector, Bhadelia, Sonti [20] found no effect of occupation type. However, Bracewell, Campbell [21], Chiu, Black [25], and Gudgeon, Wells [27] all found that physicians had higher associations with presenteeism than other health care workers such as nurses, assistants and students. However, it did not seem to matter if participants had professional or clinical status [25]. In addition, those who worked in a hospital setting as opposed to long-term care settings also had higher levels of presenteeism.

##### Experience

Four studies of mixed quality looked at the experience level of participants on presenteeism. No significant associations were found between length of time in the job or profession [24, 25], or the training year of medical students [28, 33] with presenteeism.

##### Working hours

Two studies looked at the number of hours worked per week [21, 26] (e.g. full time or on a part time employment) and found no significant associations with presenteeism.

##### Patient type

Two studies of mixed quality looked at whether the type of patient that healthcare workers cared for was a factor associated with presenteeism and found no associations [25, 34].

##### Other

Other factors related to employment included job satisfaction, and the amount of work left undone if absent [21], none of which showed any significant associations with presenteeism. Participant perceptions of the infection control measures at their institution were associated with presenteeism, with those who thought there was poorer control showing higher levels of presenteeism [31]. Rousculp, Johnston [37] also identified that those who could not work from home when sick had higher levels of presenteeism. However, all these factors have only been studied once, therefore our ability to draw conclusions from this is limited.

## Discussion

In our review, the overall prevalence of infectious illness presenteeism ranged from 35 to 97%. Although a very broad range of estimates, even the lower end of this range is troubling and is likely to result in increased transmission of infection in a workplace or school. This range is in line with previous studies of presenteeism prevalence relating to ill health in general [1, 41, 42]. Our review found that rates of presenteeism were generally higher in health and social care workers, which matched the results from existing literature [4, 5]. Again, given the vulnerable populations these groups interact with, this is a source of some concern.

Reported reasons for infectious illness presenteeism could be grouped into three main themes concerning organisational characteristics, job characteristics and personal reasons. Common organisational factors included policy regarding sick leave, with a lack of flexible sick leave and strict attendance control protocol appearing to stimulate presenteeism in employees [43, 44]. Similarly, applying a policy of sick leave allowance may encourage employees to save their allowance for family emergencies, again leading to an increase in presenteeism [45]. Other organisational factors concerned the perception of a presenteeism culture. Pressure from organisations, supervisors and colleagues to work while ill, and the urge to maintain a positive relationship with co-workers were often given as reasons. It is possible that employees were reluctant to call in sick due to the fear of receiving negative comments from colleagues or to avoid creating tension with supervisors who may question the legitimacy of their sickness [46]. Fear of receiving disciplinary actions was also reported, yet it is important to note that punishments were mainly anticipated, and many respondents may not have been penalized.

In terms of job characteristics, a perceived ‘lack of cover’ was widely reported, especially in respondents who were health care professionals. Employees in this sector might find it more difficult to find backups due to their specialized roles or due to general understaffing in the workplace [32]. Because of these highly specialised roles another related reason concerned professionalism, and the fact it was their duty to provide care to patients and not to disrupt this.

Personal reasons mentioned included “fear of increasing burden on others”, and to avoid feeling guilty [47]. Moreover, many respondents were unsure about the threshold of taking sick leaves since they were uncertain if their symptoms were severe enough for sickness absence and thought they were not infectious. It is interesting that many physicians also reported similar reasons, despite their relative expertise in this area. This could be explained by the reluctance of physicians to recognize sickness in themselves and their incongruent perceptions of illness, as they may compare their illnesses with their patients’ and conclude they are not sick enough to stay at home [24, 38].

The risk factors tested for associations with infectious illness presenteeism could be grouped into four main themes: sociodemographic, health, influenza-related behaviour and job characteristics.

For sociodemographic factors, we found inconclusive evidence for the role of gender. This seems to reflect what is found in the general presenteeism literature in which some studies find males tend to exhibit more presenteeism [48, 49], and others females [50, 51]. There was some indication that age was associated with presenteeism, with those that were younger showing higher rates of presenteeism, again reflecting findings in the general presenteeism literature [52, 53] however the results were overall inconclusive.

For health, participants’ general health or prevalence of chronic conditions such as asthma or diabetes was not associated with presenteeism, but having a healthy immune system was. This is at first sight surprising as previous evidence suggests it is those with poorer general health that are at risk of sickness presence [54–56]. Employees with poor health may believe that they are compelled to work due to the time off that they already have taken [57]. Alternatively, it may be that those who feel they have a healthy immune system believe they can fend of infectious illnesses and therefore not be a risk of transmission to colleagues.

Not surprisingly past influenza-related behaviours and intentions show some associations with infectious illness presenteeism. As is well documented in health behaviour theory [58] peoples past behaviours and intentions predict their future behaviour, and in this case those who have had a higher number of presenteeism days in the past and intend to go to work with an infectious illness do indeed have higher rates of presenteeism.

For many of the variables which fell under job characteristics and were assessed for associations with presenteeism, the evidence base was weak. Variables such as patient type and working hours had been assessed in two studies showing no associations, but again this limited evidence base is not enough to justify any robust conclusions. By far the most important factor coming under job characteristics was occupation type. We found that working in the health care sector was a risk factor for infectious illness presenteeism, which was in line with pervious literature [54]. Different studies showed that health care professionals, especially physicians, generally reported more sickness presence [19, 21, 25, 27]. The daily tasks of health care workers usually involve providing care services, and the relationships between these employees and their clients or students can play a crucial role in job outcomes. It is believed that such relationships would predispose employees to sickness presence [54].

### Quality of included studies

The majority of included studies were cross-sectional, and therefore causal relationships could not be established. Although the overall quality of studies was poor with many studies at a high risk of sampling and non-response bias, we did not find an obvious trend for high quality studies to report more significant results compared to low quality studies. Contrasting results were often found and acknowledged by authors; however, attempts were seldom made to explain such inconsistencies. Some frequently reported reasons, such as lack of cover were often not tested in quantitative studies. Those reasons could be adapted and tested as variables in quantitative studies to provide a more comprehensive result. The exclusive use of self-reported data to measure presenteeism was another limitation. Although it is understandable that objective data is hard to obtain in this situation, additional questions that provide more details of sickness presence may be a better measure.

### Quality of this review

This comprehensive review explored the prevalence, reasons and risk factors for infectious illness presenteeism. A major strength is that two independent reviewers went through the screening process for one of the databases, meaning subjective views and human errors were minimized. This process was then followed for the remaining databases by a third independent reviewer and any inconsistencies discussed. However, this present review suffers from various limitations. Firstly, although we searched three large databases, it is possible that we missed some articles that would have fit our criteria. Secondly a large proportion of studies were excluded for not being clear that they were measuring presenteeism because of an infectious illness. It is possible that some of these studies were incorrectly excluded. Thirdly, the quality of our review may be limited by publication bias. It is plausible that some studies with non-significant results were not published. It is also worth noting that many of our included studies had health care professionals as their target populations, as such the findings of this review may be more representative of the healthcare sector than other organisational settings. Additionally, reasons for and variables tested for associations with infectious illness presenteeism were grouped into common themes and presented in our results to ease interpretation, but in spite of our best efforts in clustering them, clear differentiation was not always possible.

### Implications for research and practice

The results show that presenteeism is common in employees, leading to an increased risk of disease transmission. Longitudinal studies are now needed to establish causality among variables and provide more substantial evidence regarding risk factors for infectious illness presenteeism which can be subsequently addressed in interventions. It is also important for standardised and objective measurements of sickness presence to be formulated which will help to increase the consistency and comparability of research in this field. Risk factors that provided contrasting results, such as gender, age, and those with a small evidence base such as dependents and various job-related factors should be replicated to verify associations. Other sociodemographic variables such as ethnicity and education were not a focus in existing literature, but further study in this area would be useful. Additionally, many of the existing studies solely focused on infectious illness presenteeism in health care staff, studies should also explore this in other industries and non-workplace environments such as schools and nursery’s in order to explore specific risk factors in different settings. The lack of studies that have tested interventions to reduce infectious illness presenteeism was also striking. As evidence for the risk factors of presenteeism begins to grow, it will be important to use these to develop intervention programmes that address these and cater to the different needs of health sector vs non-health sector organisations and schools.

From the results reported here it, a fruitful avenue for such interventions may be for organisations to promote the legitimacy of taking sick leave and emphasise the negative impacts of presenteeism. At the organisational level, specific job-related risk factors, such as lack of cover, should be identified so that counter measures can be developed. Since many employees are unsure about the threshold for taking sick leave, clear guidelines should also be given regarding what to do when they are sick. To minimise external pressure, it is also crucial for bosses to cultivate an organisational culture that emphasises the importance and benefits of taking sick leave and recognises the potentially hazardous impacts of sickness presence, especially the increased risks of spreading infectious diseases to other employees. At the individual level, workload should be properly managed and monitored. Although reducing workload is not always achievable, skills, resources and techniques can be enhanced to help workers cope with job demands [56]. Supervisors and managers should act as role models and be supportive of workers who require sick leave. Having a supportive environment could encourage illness disclosure and reduce the negative feelings associated with absenteeism, such as guilt from burdening others [59].

Even though the above-mentioned strategies may not show immediate results, such integrated and tailored approaches could provide long term economic, social and personal benefits from reducing presenteeism. In particular focusing on the management of presenteeism now, rather than during a pandemic [60], puts us in a stronger position for when a new pandemic does happen. Developing policies and interventions designed to reduce presenteeism in different organisations, as well as schools and nurseries, will facilitate the rapid implementation of strategies to mitigate the impacts of a new pandemic when the risks are greater to the working population and children.

## Conclusion

This review analysed data on the prevalence, reported reasons and risk factors for infectious illness presenteeism, and provides insights for researchers and practitioners. Our results indicate that infectious illness presenteeism is common in organisations. While some variables were found to be associated with working with a suspected infectious illness, such as occupation type and influenza-related behaviour, many others showed inconsistent results. Given the uncertainties and additional risks of disease transmission, future research is needed, which should include the development and implementation of interventions.

## Additional file

Additional file 1: Search strategy. Example of search strategy used in MEDLINE (DOCX 13 kb)

## Data Availability

All data is contained within the published manuscript.

## Abbreviations

HCPs: Health care professionals
HCWs: Health care workers
ILI: Infectious like illness
M: Mean
Me: Median
MMAT: Mixed methods appraisal tool
nr: Not reported
RTI: Respiratory tract infection
