# Supplementary material for: A systematic review of infectious illness Presenteeism: prevalence, reasons and risk factors
Source: BMC Public Health. 2019 Jun 21;19:799. doi: 10.1186/s12889-019-7138-x (PMC6588911; doi:10.1186/s12889-019-7138-x)
Supplement: Supplementary file 1 — Search strategy. Example of search strategy used in MEDLINE (DOCX 13 kb) [file 12889_2019_7138_MOESM1_ESM.docx]

## Search strategy

MEDLINE

1 exp Diarrhea/ OR diarrhea.mp. OR diarrhoea.mp.

2 exp Vomiting/ OR vomit.mp.

3 rash.mp. OR exp Exanthema/

4 influenza-like-illness.mp.

5 ILI.mp.

6 (Hand foot and mouth disease).mp.

7 measles.mp. OR exp Measles/ OR Measles virus/

8 mumps.mp. OR exp Mumps/

9 exp Rubella/ OR rubella.mp.

10 exp Chickenpox/ OR chickenpox.mp.

11 exp Hemorrhagic Fever, Ebola/ OR ebola.mp.

12 exp Plague/ OR plague.mp.

13 meningitis.mp. OR exp Meningitis/ OR exp Meningitis, Viral/

14 exp Tuberculosis/ OR tuberculosis.mp.

15 gastroenteritis.mp. OR exp Gastroenteritis/

16 flu.mp. OR exp Influenza, Human/

17 pandemic.mp. OR exp Pandemics/

18 influenza.mp. OR exp Influenza, Human/

19 disease outbreak.mp. OR exp Disease Outbreaks/

20 infectious.mp.

21 contagious.mp.

22 severe acute respiratory syndrome.mp. OR exp Severe Acute Respiratory Syndrome/

23 exp Hemorrhagic Fevers, Viral/ OR Viral haemorrhagic fever.mp.

24 1 OR 2 OR 3 OR 4 OR 5 OR 6 OR 7 OR 8 OR 9 OR 10 OR 11 OR 12 OR 13 OR 14 OR 15 OR 16 OR 17 OR 18 OR 19 OR 20 OR 21 OR 22 OR 23

25 attendence.mp.

26 return to work.mp. OR exp Return to Work/

27 sick leave.mp. OR exp Sick Leave/

28 25 OR 26 OR 27

29 presenteeism.mp. OR exp Presenteeism/

30 24 AND 28

31 29 OR 30

32 limit 31 to english language

33 limit 32 to “review articles”

34 32 not 33
